# Supplementary material for: Differences in access to water, sanitation, and hygiene facilities among residents of Korail Slum, Bangladesh, during normal vs. water-logging situations
Source: PLoS One. 2025 Sep 19;20(9):e0332534. doi: 10.1371/journal.pone.0332534 (PMC12449000; doi:10.1371/journal.pone.0332534)
Supplement: S1 File — (PDF) [file pone.0332534.s001.pdf]

Original Questionnaire

The effect of water logging on water, sanitation and hygiene (WASH) conditions in Korail Slums in Dhaka, Bangladesh.

SL	Questions	Answers (Please tick)	Code	Skip
	Unique ID			
	Date			
**A**	**Demographic characteristics.**			
a1	What is your age? (Please specify in years)			
a2	What is your gender? (select_one)	Male	1	
		Female	2	
		Third gender	3	
		Others (Specify)	77	
		Refuse to answer	99	
a3	What is your highest level of education completed? (select_one)	Never went to school	1	
		Primary school	2	
		Junior high school	3	
		High school	4	
		SSC/equivalent	5	
		HSC/equivalent	6	
		Vocational certificate	7	
		Degree/equivalent	8	
		Masters/equivalent or higher	9	
		Others (Specify)	77	
		Not sure	88	
		Refuse to answer	99	
a4	What is your marital status? (select_one)	Single/Never married	1	
		Married	2	
		Divorced	3	
		Widowed/Separated	4	
		Others (Specify)	77	
		Refuse to answer	99	
a5	What is your occupation? (select_one)	Rickshaw/Van/Cart puller	1	
		Garments worker	2	
		Transport worker	3	
		Construction worker	4	
		Hotel worker	5	
		Driver	6	
		Business	7	
		Service	8	
		Cottage industry	9	
		Hawker	10	
		Agri-labour	11	
		Porter/Day labour	12	
		Servant/Maid servant	13	
		Student	14	
		Not working/Disabled	15	

Original Questionnaire

SL	Questions	Answers (Please tick)	Code	Skip
		Retired/ homemaker	16	
		Housewife	17	
		Other (specify)	77	
		Refuse to answer	99	
a6	What is your religion? (select_one)	Islam	1	
		Hinduism	2	
		Buddhism	3	
		Christianity	4	
		No religion	5	
		Other (specify)	77	
		Refuse to answer	99	
a7	Household monthly income? (select_one)	No more than 5,000 Taka	1	
		5,001 to 10,000 Taka	2	
		10,001 to 20,000 Taka	3	
		20,001 to 30,000 Taka	4	
		30,001 to 40,000 Taka	5	
		40,001 to 50,000 Taka	6	
		More than 50,000 Taka	7	
		Not sure / Uncertain / Don't know	88	
		Refuse to answer	99	
a8	How many members are there in your household, including yourself? (Please specify the number; if refuse to answer, write 99)			
a9	What is your relation with the head of household? (select_one)	Respondent is the head of household	1	
		Wife/husband/partner	2	
		Son/daughter (unmarried)	3	
		Son/daughter (married)	4	
		Son/daughter-in-law	5	
		Grandchild	6	
		Parent/Parent-in-law	7	
		Sibling	8	
		Other relatives	9	
		Domestic assistant/non-relative	10	
		Other (specify)	77	
		Not sure	88	
		Refuse to answer	99	
a10	Ownership of housing structure? (select_one)	Owned house	1	
		Rented house	2	
		Government provided house	3	
		Other (specify)	77	
		Refuse to answer	99	
a11	Does your household have? (select_multiple)	Electricity	1	
		Solar electricity	2	
		Television	3	

Original Questionnaire

SL	Questions	Answers (Please tick)	Code	Skip
		Refrigerator	4	
		Almira/wardrobe	5	
		A sofa set	6	
		Table/Chair	7	
		Electric fan	8	
		DVD/CD player	9	
		Water filter	10	
		Washing machine	11	
		Water pump	12	
		IPS/generator	13	
		Air conditioner	14	
		None of above	88	
		Refuse to answer	99	
a13	Does any member of this household own? (select_multiple)	Car/truck/microbus	1	
		Auto bike/CNG/tempo	2	
		Motorcycle/motor scooter	3	
		Rickshaw/Van	4	
		Bicycle	5	
		Smart mobile phone	6	
		Normal mobile phone	7	
		Computer/Laptop	8	
		None of above	88	
		Refuse to answer	99	
**B**	**Access to Water, Sanitation, and Hygiene**			
**B1**	**Non-waterlogging periods**	**Prompt: "Generally, when you live here and there is NO water logging..."**		
**b1w**	**Water during non-logging periods**			
b1w1	What is the main source of drinking water for members of your household? (select_one) (If unclear, probe to identify the place from which members of this household most often collect drinkink water-collection point)	Piped water-Piped into dwelling	1	
		Piped water-Piped into compound, yard or plot	2	
		Piped water-Piped to neighbour	3	
		Piped water-Public tap/standpipe	4	
		Piped water-Borehole or tubewell	5	
		Dug well-Protected well	6	
		Dug well-Unprotected well	7	
		Water from spring-Protected spring	8	
		Water from spring-Unprotected spring	9	
		Rainwater collection	10	
		Delivered water-Tanker-truck	11	
		Delivered water-Cart with small tank/drum	12	
		Water kiosk	13	
		Packaged water-Bottled water	14	
		Packaged water-Sachet water	15	

Original Questionnaire

SL	Questions	Answers (Please tick)	Code	Skip
		Surface water (river, stream, dam, lake, pond, canal, irrigation channel)	16	
		Other (specify)	77	
		Refuse to answer	99	
b1w2	Do you or any other member of this household do anything to the water to make it safer to drink? (select_one)	Yes	1	b1w2a
		No	2	
		Don’t know	88	
		Refuse to answer	99	
b1w2a	What do you usually do to make the water safer to drink? (select_multiple) (Probe: Anything else? Record all methods mentioned)	Boil	1	
		Add bleach/chlorine	2	
		Strain it through a cloth	3	
		Use water filter (ceramic, sand, composite, etc.)	4	
		Solar disinfrction	5	
		Let it stand and settle	6	
		Other (specify)	77	
		Don’t know	88	
		Refuse to answer	99	
b1w3	What is the main source of water used by members of your household for other purposes, such as cooking and hand washing? (select_one)	Piped water-Piped into dwelling	1	
		Piped water-Piped into compound, yard or plot	2	
		Piped water-Piped to neighbour	3	
		Piped water-Public tap/standpipe	4	
		Piped water-Borehole or tubewell	5	
		Dug well-Protected well	6	
		Dug well-Unprotected well	7	
		Water from spring-Protected spring	8	
		Water from spring-Unprotected spring	9	
		Rainwater collection	10	
		Delivered water-Tanker-truck	11	
		Delivered water-Cart with small tank/drum	12	
		Water kiosk	13	
		Packaged water-Bottled water	14	
		Packaged water-Sachet water	15	
		Surface water (river, stream, dam, lake, pond, canal, irrigation channel)	16	
		Other (specify)	77	
		Refuse to answer	99	
b1w4	Where is that water collected from? (select_one	In own dwelling	1	
		In own block/plot	2	b1w5
		Elsewhere	3	b1w5
		Refuse to answer	99	
b1w5	How long does it take to go there, get water, and come back? (In minutes, If not collected=00, Don't know=88, Reffuse to answer=99)			

Original Questionnaire

SL	Questions	Answers (Please tick)	Code	Skip
b1w6	Who usually goes to this source to collect the drinking water for your household? (select_one)	Adult women (over 15 years age)	1	
		Adult men (over 15 years age)	2	
		Female child (age 15 years below)	3	
		Male child (age 15 years below)	4	
		Other (specify)	77	
		Refuse to answer	99	
b1w7	Who usually goes to this source to collect the non-drinking water for your household? (select_one)	Adult women (over 15 years age)	1	
		Adult men (over 15 years age)	2	
		Female child (age 15 years below)	3	
		Male child (age 15 years below)	4	
		Other (specify)	77	
		Refuse to answer	99	
b1w8	In the last month, has there been any time when your household did not have sufficient quantities of drinking water when needed? (select_one)	Yes, at lest once	1	
		No, always sufficient	2	
		Don’t know	88	
		Refuse to answer	99	
b1w9	What was the main reason that you were unable to access drinking water in sufficient quantities when needed? (select_one)	Not functional	1	
		Water not available from source	2	
		Water to expensive	3	
		Source not accessible	4	
		Other (specify)	77	
		Don’t know	88	
		Refuse to answer	99	
b1w9a	If water source is not functioning and any of your neighbors can not fix it, who would you most often report the problem? (select_one)	Landlord/mastan	1	
		D-WASA (central government)	2	
		DPHE (central government)	3	
		Private company or individual	4	
		Mosque/school/clinic/community institution	5	
		NGO	6	
		Community leader	7	
		No one	8	
		Not applicable (use surface water)	9	
		Other (specify)	77	
		Don’t know	88	
		Refuse to answer	99	
b1w10	Do other households use this water source? (select_one)	Yes	1	
		No	2	
b1w11	How many Households collect water from the same water source? (Number of Households)			

Original Questionnaire

SL	Questions	Answers (Please tick)	Code	Skip
b1w12	On average, how long do you/the household member have to wait in the queue to get water? (minutes, Don't know=88, Not applicable (do not share water source with anyone=99)			
b1w13	How long has your household been using the water source? (in months; if more than 5 years, write 61; if don't know, write 88; if not applicable or refuse to answer, write 99)			
b1w14	Who most frequently manages this water source? (select_one)	Own	1	
		Landlord/mastan	2	
		D-WASA (central government)	3	
		DPHE (central government)	4	
		Private company or individual	5	
		Mosque/school/clinic/community institution	6	
		NGO	7	
		Community leader	8	
		Not applicable (use surface water)	9	
		Other (specify)	77	
		Don’t know	88	
		Refuse to answer	99	
b1w15	Who installed this water source? (select_one) (If multiple installers, choose the organization or group with the biggest role)	Own	1	
		Landlord/mastan	2	
		D-WASA (central government)	3	
		DPHE (central government)	4	
		Private company or individual	5	
		Mosque/school/clinic/community institution	6	
		NGO	7	
		Community leader	8	
		Not applicable (use surface water)	9	
		Other (specify)	77	
		Don’t know	88	
		Refuse to answer	99	
b1w16	Typically, how many hours a day are you able to obtain water from this source? (how many hours)			
b1w17	Do you pay to use your main drinking water source? (select_one)	Yes	1	b1w18
		No	2	
		Don’t know	88	
		Refuse to answer	99	
b1w18	How are you charged to access to main drinking water source? (select_one) (if the method changes, choose the	Per use	1	
		Fixed amount per week	2	
		Fixed amount per month	3	
		Fixed amount per year	4	

SL	Questions	Answers (Please tick)	Code	Skip
	method that you use most often)	Other (specify)	77	
		Don't know	88	
		Refuse to answer	99	
b1w19	Approximately, how much did you pay for water each time you made a payment? (in BDT, if no payment, write 0; if don't know, write 888; if not applicable, write 999)			
b1w20	To whom do you pay this amount? (select_one)	Landlord/mastan	1	
		D-WASA (central government)	2	
		Private company or individual	3	
		Mosque/school/clinic/community institution	4	
		NGO	5	
		Community leader	6	
		Tranker truck manager	7	
		Water vendor	8	
		Other (specify)	77	
		Don't know	88	
		Refuse to answer	99	
**b1s**	**Sanitation during non-logging periods**			
b1s1	What kind of toilet facility do members of your household usually use? (select_one) -If 'Flush' or 'Pour flush', probe: Where does it flush to?	**Flush/pour flush**-Flush to piped sewer system	1	
		**Flush/pour flush**-Flush to septic tank	2	
		**Flush/pour flush**-Flush to pit latrine	3	
		**Flush/pour flush**-Flush to open drain	4	
		**Flush/pour flush**-Flush to don't know where	5	
		**Dry pit latrines**-Pit latrine with slab	6	
		**Dry pit latrines**-Pit latrine without slab / Open pit	7	
		**Composting toilets-**Twin pit with slab	8	
		**Composting toilets**-Twin pit without slab	9	
		**Composting toilets**-Other composting toilet	10	
		**Bucket**-Container based sanitation	11	
		**Bucket**-Hanging toilet / hanging latrine	12	
		No facility / Bush / Field	13	
		Others (Specify)	77	
		Refuse to answer	99	
b1s2	Do you usually (every day or almost every day) share this	Yes	1	
		No	2	

Original Questionnaire

SL	Questions	Answers (Please tick)	Code	Skip
	facility with others who are not members of your household? (select_one)	Don't know	88	
		Refuse to answer	99	
b1s3	How many household use this toilet facility? (If no other HH write=1, Don't know=88)			
b1s4	Where is this toilet facility located? (select_one)	In own dwelling	1	
		In own block/plot	2	
		Elsewhere	3	
		Refuse to answer	99	
b1s5	On average, how long do you/the household member have to wait in the queue to use toilet? (minutes, if don't know, write 88; if not applicable or on premise or not shared with others, write 99)			
b1s6	Approximately, how much did you pay each time you use the toilet? (in BDT, if no payment, write 0; if don't know, write 888; if not applicable or on premise or not shared with others, write 999)			
b1s7	To whom do you pay this amount? (select_one)	Landlord/mastan	1	
		D-WASA (central government)	2	
		DPHE (central government)	3	
		Private company or individual	4	
		Mosque/school/clinic/community institution	5	
		NGO/CBO	6	
		Community leader	7	
		Other (specify)	77	
		Don't know	88	
		Refuse to answer	99	
b1s8	In the past year, was there ever a time when your household's primary toilet facility was not functional? (select_one)	Yes	1	
		No	2	
		Don't know	88	
		Refuse to answer	99	
b1s8a	How many days was household's primary toilet facility not functional? (Days, Don't know=88)			
b1s8b	If toilet facility is not functioning and any of your neighbors can not fix it, who would you report the problem? (select_one)	Landlord/mastan	1	
		D-WASA (central government)	2	
		DPHE (central government)	3	
		Private company or individual	4	
		Mosque/school/clinic/community institution	5	
		NGO/CBO	6	
		Community leader	7	

Original Questionnaire

SL	Questions	Answers (Please tick)	Code	Skip
		Other (specify)	77	
		Don't know	88	
		Refuse to answer	99	
b1s9	Do you have to pay to use this toilet? (select_one)	Yes	1	
		No	2	
		Don't know		
		Refuse to answer	99	
b1s10	How are you charged for access to this toilet facility? (select_one)	Per use	1	
		Fixed amount per week	2	
		Fixed amount per month	3	
		Fixed amount per year	4	
		Other (specify)	77	
		Don't know	88	
		Refuse to answer	99	
b1s11	Is it safe for adult males to use this toilet facility at night? (select_one)	Yes	1	
		No	2	
		Don't know	88	
		Refuse to answer	99	
b1s12	Is it safe for adult females to use this toilet facility at night? (select_one)	Yes	1	
		No	2	
		Don't know	88	
		Refuse to answer	99	
b1s13	Is it safe for children to use this toilet facility at night? (select_one)	Yes	1	
		No	2	
		Don't know	88	
		Refuse to answer	99	
**b1h**	**Hygiene practices during non-logging periods**			
b1h1	Where do you and other member of your household most often wash your hands? (select_one)	Fixed facility reported (sink/tap)-In dwelling	1	
		Fixed facility reported (sink/tap)-In yard/plot	1	
		Mobile object reported (bucket/jug/kettle)	2	
		No handwashing place in dwelling/yard/plot	3	
		Others (Specify)	77	
		Refuse to answer	99	
b1h2	What do you usually wash your hands? (select_multiple)	Don't wash hand	0	
		Only water	1	
		Soap	2	
		Detergent	3	
		Ash	4	
		Mud/sand	5	
		Others (Specify)	77	
		Not sure	88	
		Refuse to answer	99	

Original Questionnaire

SL	Questions	Answers (Please tick)	Code	Skip
b1h3	Do you have any soap or detergent in your household for washing hands? (select_one)	Yes	1	
		No	2	
		Not applicable/I don't know	88	
		Refuse to answer	99	
b1h4	What are the important occasions when people need to wash their hands? (select_multiple). (No prompting; wait to hear the response then check the appropriate boxes).	After going to the toilet	0	
		After attending to a child who has defecated	1	
		Before preparing food	2	
		Before feeding a child	3	
		Before eating	4	
		None of the above mentioned	5	
		I don't know	88	
		Refuse to answer	99	
b1h5	Within the past week, How often do you wash your hands with water and bar soap / detergent / liquid soap after going to the toilet? (select_one)	Always (100% of the time)	1	
		Mostly (80-100%)	2	
		Frequently (60-79.99%)	3	
		Sometimes (40-59.99%)	4	
		Seldom (20-39.99%)	5	
		Never or almost never (0-19.99%	6	
		Not applicable	88	
b1h6	Within the past week, How often do you wash your hands with water and bar soap / detergent / liquid soap after changing a child's diaper with feces? (select_one)	Always (100% of the time)	1	
		Mostly (80-100%)	2	
		Frequently (60-79.99%)	3	
		Sometimes (40-59.99%)	4	
		Seldom (20-39.99%)	5	
		Never or almost never (0-19.99%	6	
		Not applicable (no diaper-wearing child in the househld)	88	
b1h7	Within the past week, How often do you wash your hands with water and bar soap / detergent / liquid soap before preparing food? (select_one)	Always (100% of the time)	1	
		Mostly (80-100%)	2	
		Frequently (60-79.99%)	3	
		Sometimes (40-59.99%)	4	
		Seldom (20-39.99%)	5	
		Never or almost never (0-19.99%	6	
		Not applicable	88	
b1h8	Within the past week, How often do you wash your hands with water and bar soap / detergent / liquid soap before feeding a child? (select_one)	Always (100% of the time)	1	
		Mostly (80-100%)	2	
		Frequently (60-79.99%)	3	
		Sometimes (40-59.99%)	4	
		Seldom (20-39.99%)	5	
		Never or almost never (0-19.99%	6	
		Not applicable (no diaper-wearing child in the househld)	88	
b1h9		Always (100% of the time)	1	

Original Questionnaire

SL	Questions	Answers (Please tick)	Code	Skip
	Within the past week, How often do you wash your hands with water and bar soap / detergent / liquid soap before eating? (select_one)	Mostly (80-100%)	2	
		Frequently (60-79.99%)	3	
		Sometimes (40-59.99%)	4	
		Seldom (20-39.99%)	5	
		Never or almost never (0-19.99%	6	
		Not applicable	88	
b1h10	How do you usually dispose household waste from your house? (select_one)	In open pit	1	
		Discard in the yard/within house	2	
		Discard outside house	3	
		Others (Specify)	77	
		I don't know	88	
		Refuse to answer	99	
**b2**	**WASH during water logging periods**	**Prompt: "Generally, when you live here and there is water logging..."**		
b2d	Water logging			
b2d1	Has your community ever experienced water logging in the past year?? (select_one)	Yes	1	
		No	2	
		I don't know	88	
		Refuse to answer	99	
b2d1a	Within the past 12 months, how many times did this community experience water logging? (select_one)	1 time	1	
		2-3 times	2	
		4-5 times	3	
		More than 5 times	4	
		Not sure / Don't know	88	
		Refuse to answer	99	
b2d1b	During the latest water logging events, how long did the water remain stagnant in your area? (select_one)	Less than 10 hours	1	
		More than 10 hours	2	
		1 days	3	
		2-4 days	4	
		More than 4 days	5	
		Not sure / Don't know	88	
		Refuse to answer	99	
**b2w**	**Water during logging**			
b2w1	During the latest water logging period, did you and your family use the same main source of drinking water as when there was non-logging?	Yes	1	
		No	2	B2w4
b2w2	What is the main source of drinking water for members of your household? (select_one) (If unclear, probe to identify the place from which members of this household collect drinkink water-	Piped water-Piped into dwelling	1	
		Piped water-Piped into compound, yard or plot	2	
		Piped water-Piped to neighbour	3	
		Piped water-Public tap/standpipe	4	
		Piped water-Borehole or tubewell	5	

Original Questionnaire

SL	Questions	Answers (Please tick)	Code	Skip
	collection point during water logging)	Dug well-Protected well	6	
		Dug well-Unprotected well	7	
		Water from spring-Protected spring	8	
		Water from spring-Unprotected spring	9	
		Rainwater collection	10	
		Delivered water-Tanker-truck	11	
		Delivered water-Cart with small tank/drum	12	
		Water kiosk	13	
		Packaged water-Bottled water	14	
		Packaged water-Sachet water	15	
		Surface water (river, stream, dam, lake, pond, canal, irrigation channel)	16	
		Other (specify)	77	
		Refuse to answer	99	
b2w3	Do you or any other member of this household do anything to the water to make it safer to drink? (select_one)	Yes	1	
		No	2	
		Don’t know	88	
		Refuse to answer	99	
b2w3a	What do you usually do to make the water safer to drink? (select_multiple) (Probe: Anything else? Record all methods mentioned)	Boil	1	
		Add bleach/chlorine	2	
		Strain it through a cloth	3	
		Use water filter (ceramic, sand, composite, etc.)	4	
		Solar disinfrction	5	
		Let it stand and settle	6	
		Other (specify)	77	
		Don’t know	88	
		Refuse to answer	99	
b2w4	During the latest water logging period, did you and your family use the same main source of your household for other purposes water as when there was non-logging?	Yes	1	
		No	2	B2w10
b2w5	What is the main source of water used by members of your household for other purposes, such as cooking and hand washing? (select_one)	Piped water-Piped into dwelling	1	
		Piped water-Piped into compound, yard or plot	2	
		Piped water-Piped to neighbour	3	
		Piped water-Public tap/standpipe	4	
		Piped water-Borehole or tubewell	5	
		Dug well-Protected well	6	
		Dug well-Unprotected well	7	
		Water from spring-Protected spring	8	
		Water from spring-Unprotected spring	9	
		Rainwater collection	10	

Original Questionnaire

SL	Questions	Answers (Please tick)	Code	Skip
		Delivered water-Tanker-truck	11	
		Delivered water-Cart with small tank/drum	12	
		Water kiosk	13	
		Packaged water-Bottled water	14	
		Packaged water-Sachet water	15	
		Surface water (river, stream, dam, lake, pond, canal, irrigation channel)	16	
		Other (specify)	77	
		Refuse to answer	99	
b2w6	Where is that water collected from? (select_one)	In own dwelling	1	
		In own block/plot	2	
		Elsewhere	3	
		Refuse to answer	99	
b2w7	How long does it take to go there, get water, and come back? (In minutes, If not collected=00, Don't know=88)			
b2w8	Who usually goes to this source to collect the water for your household?	Adult women (over 15 years age)	1	
		Adult men (over 15 years age)	2	
		Female child (age 15 years below)	3	
		Male child (age 15 years below)	4	
		Other (specify)	77	
		Refuse to answer	99	
b2w9	Who usually goes to this source to collect the non-drinking water for your household? (select_one)	Adult women (over 15 years age)	1	
		Adult men (over 15 years age)	2	
		Female child (age 15 years below)	3	
		Male child (age 15 years below)	4	
		Other (specify)	77	
		Refuse to answer	99	
b2w10	During the water logging period, was there any time when your household did not have sufficient quantities of drinking water when needed? (select_one)	Yes, at lest once	1	
		No, always sufficient	2	
		Don’t know	88	
		Refuse to answer	99	
b2w11	What was the main reason that you were unable to access water in sufficient quantities when needed?	Not functional	1	
		Water not available from source	2	
		Water to expensive	3	
		Source not accessible	4	
		Other (specify)	77	
		Don’t know	88	
		Refuse to answer	99	
b2w11a	During the latest water logging period, If water source is not functioning and any of your neighbors can not fix it, who would you report the problem? (select_one)	Landlord/mastan	1	
		D-WASA (central government)	2	
		DPHE (central government)	3	
		Private company or individual	4	
		Mosque/school/clinic/community institution	5	

Original Questionnaire

SL	Questions	Answers (Please tick)	Code	Skip
		NGO	6	
		Community leader	7	
		No one	8	
		Not applicable (use surface water)	9	
		Other (specify)	77	
		Don’t know	88	
		Refuse to answer	99	
b2w12	Do other households use this water source? (select_one)	Yes	1	
		No	2	
b2w13	How many Households collect water from the same water source? (Number of Households)			
b2w14	On average, how long do you/the household member have to wait in the queue to get water? (minutes, Don't know=88, Not applicable (do not share water source with anyone=99)			
b2w15	Who most frequently manages this water source? (select_one)	Own	1	
		Landlord/mastan	2	
		D-WASA (central government)	3	
		DPHE (central government)	4	
		Private company or individual	5	
		Mosque/school/clinic/community institution	6	
		NGO	7	
		Community leader	8	
		Not applicable (use surface water)	9	
		Other (specify)	77	
		Don’t know	88	
		Refuse to answer	99	
b2w16	Who installed this water source? (select_one) (If multiple installers, choose the organization or group with the biggest role)	Own	1	
		Landlord/mastan	2	
		D-WASA (central government)	3	
		DPHE (central government)	4	
		Private company or individual	5	
		Mosque/school/clinic/community institution	6	
		NGO	7	
		Community leader	8	
		Not applicable (use surface water)	9	
		Other (specify)	77	
		Don’t know	88	
		Refuse to answer	99	

Original Questionnaire

SL	Questions	Answers (Please tick)	Code	Skip
b2w17	Typically, how many hours a day are you able to obtain water from this source? (how many hours)			
b2w18	Do you pay to use your main drinking water source? (select_one)	Yes	1	
		No	2	
		Don't know	88	
		Refuse to answer	99	
b2w19	During water logging, was the water from this source not available for at least one full day?	Yes	1	
		No	2	
		Don't know	88	
		Refuse to answer	99	
b2w20	What was the main reason that you were unable to access water in sufficient quantities when needed?	Not functional	1	
		Water not available from source	2	
		Water to expensive	3	
		Source not accessible	4	
		Other (specify)	77	
		Don't know	88	
		Refuse to answer	99	
b2w21	To whom do you pay this amount? (select_one)	Landlord/mastan	1	
		D-WASA (central government)	2	
		Private company or individual	3	
		Mosque/school/clinic/community institution	4	
		NGO	5	
		Community leader	6	
		Tranker truck manager	7	
		Water vendor	8	
		Other (specify)	77	
		Don't know	88	
		Refuse to answer	99	
**b2s**	**Sanitation logging periods**			
b2s1	During the latest water logging period, did you and your family use the same toilet facility as when there was non-logging period?	Yes		
		No		
b2s2	What kind of toilet facility do members of your household usually use during water logging? (select_one)	**Flush/pour flush**-Flush to piped sewer system	1	
		**Flush/pour flush**-Flush to septic tank	2	
		**Flush/pour flush**-Flush to pit latrine	3	
		**Flush/pour flush**-Flush to open drain	4	
		**Flush/pour flush**-Flush to don't know where	5	
		**Dry pit latrines**-Pit latrine with slab	6	

Original Questionnaire

SL	Questions	Answers (Please tick)	Code	Skip
		**Dry pit latrines**-Pit latrine without slab / Open pit	7	
		**Composting toilets-**Twin pit with slab	8	
		**Composting toilets**-Twin pit without slab	9	
		**Composting toilets**-Other composting toilet	10	
		**Bucket**-Container based sanitation	11	
		**Bucket**-Hanging toilet / hanging latrine	12	
		No facility / Bush / Field	13	
		Others (Specify)	77	
		Refuse to answer	99	
b2s3	Do you usually (every day or almost every day) share this facility with others who are not members of your household? (select_one)	Yes	1	b2s4
		No	2	
		Don’t know	88	
		Refuse to answer	99	
b2s4	How many household use this toilet facility? (If no other HH write=1, Don't know=88)			
b2s5	Where is this toilet facility located? (select_one)	In own dwelling	1	
		In own block/plot	2	
		Elsewhere	3	
		Refuse to answer	99	
b2s6	On average, how long do you/the household member have to wait in the queue to use toilet? (minutes, Not applicable (on premise/unshared with other HH=88)			
b2s7	During any of these times of water logging in the past year, was there ever a time when your household’s primary toilet facility was not functional? (select_one)	Yes	1	
		No	2	
		Don't know	88	
		Refuse to answer	99	
b2s8	During any of these times of water logging in the past year, how many days was household’s primary toilet facility not functional? (Days, Don't know=88)			
b2s9	Do you have to pay to use this toilet? (select_one)	Yes	1	b2s10
		No	2	
		Don’t know	88	
		Refuse to answer	99	
b2s10	How much do you pay for per use? (Amount, Don't know=88)			
b2a11		Yes	1	

Original Questionnaire

SL	Questions	Answers (Please tick)	Code	Skip
	Is it safe for adult males to use this toilet facility at night? (select_one)	No	2	
		Don't know	88	
		Refuse to answer	99	
b2s12	Is it safe for adult females to use this toilet facility at night? (select_one)	Yes	1	
		No	2	
		Don't know	88	
		Refuse to answer	99	
b2s13	Is it safe for children to use this toilet facility at night? (select_one)	Yes	1	
		No	2	
		Don't know	88	
		Refuse to answer	99	
**b2h**	**Hygiene practices during water-logging periods**			
b2h1	During the latest water logging period, where do you and other member of your household most often wash your hands? (select_one)	Fixed facility reported (sink/tap)-In dwelling	1	
		Fixed facility reported (sink/tap)-In yard/plot	1	
		Mobile object reported (bucket/jug/kettle)	2	
		No handwashing place in dwelling/yard/plot	3	
		Others (Specify)	77	
		Refuse to answer	99	
b2h2	During the latest water logging period, what do you usually wash your hands? (select_one)	Don't wash hand	0	
		Only water	1	
		Soap	2	
		Detergent	3	
		Ash	4	
		Mud/sand	5	
		Others (Specify)	77	
		Not sure	88	
		Refuse to answer	99	
b2h3	During the latest water logging period, do you have any soap or detergent in your household for washing hands? (select_one)	Yes	1	
		No	2	
		Not applicable/I don't know	88	
		Refuse to answer	99	
b2h4	During the latest water logging period, How often do you wash your hands with water and bar soap / detergent / liquid soap after going to the toilet? (select_one)	Always (100% of the time)	1	
		Mostly (80-100%)	2	
		Frequently (60-79.99%)	3	
		Sometimes (40-59.99%)	4	
		Seldom (20-39.99%)	5	
		Never or almost never (0-19.99%	6	
		Not applicable	88	
b2h5	During the latest water logging period, How often do you wash your hands with	Always (100% of the time)	1	
		Mostly (80-100%)	2	
		Frequently (60-79.99%)	3	

Original Questionnaire

SL	Questions	Answers (Please tick)	Code	Skip
	water and bar soap / detergent / liquid soap after changing a child's diaper with feces? (select_one)	Sometimes (40-59.99%)	4	
		Seldom (20-39.99%)	5	
		Never or almost never (0-19.99%	6	
		Not applicable (no diaper-wearing child in the househld)	88	
b2h6	During the latest water logging period, How often do you wash your hands with water and bar soap / detergent / liquid soap before preparing food? (select_one)	Always (100% of the time)	1	
		Mostly (80-100%)	2	
		Frequently (60-79.99%)	3	
		Sometimes (40-59.99%)	4	
		Seldom (20-39.99%)	5	
		Never or almost never (0-19.99%	6	
		Not applicable	88	
b2h7	During the latest water logging period, How often do you wash your hands with water and bar soap / detergent / liquid soap before feeding a child? (select_one)	Always (100% of the time)	1	
		Mostly (80-100%)	2	
		Frequently (60-79.99%)	3	
		Sometimes (40-59.99%)	4	
		Seldom (20-39.99%)	5	
		Never or almost never (0-19.99%	6	
		Not applicable (no diaper-wearing child in the househld)	88	
b2h8	During the latest water logging period, How often do you wash your hands with water and bar soap / detergent / liquid soap before eating? (select_one)	Always (100% of the time)	1	
		Mostly (80-100%)	2	
		Frequently (60-79.99%)	3	
		Sometimes (40-59.99%)	4	
		Seldom (20-39.99%)	5	
		Never or almost never (0-19.99%	6	
		Not applicable	88	
b2h9	During the latest water logging period, How do you usually dispose household waste from your house? (select_one)	In open pit	1	
		Discard in the yard/within house	2	
		Discard outside house	3	
		Others (Specify)	77	
		I don't know	88	
		Refuse to answer	99	
D	**History of receving help**			
d1	Did community members (e.g., neighbors or local organizations) help you during the last water logging? (select_one)	Yes	1	
		No	2	
		Don't know	88	
		Refuse to answer	99	
d2	What type of assistance do you receive from the community during water logging? (Multiple)	Access to clean drinking water	1	
		Temporary shelter	2	
		Food supplies	3	
		Sanitation facilities	4	
		Health services	5	

Original Questionnaire

SL	Questions	Answers (Please tick)	Code	Skip
		Financial support	6	
		I don't get any support	7	
		Other (please specify)	77	
		Don't know	88	
		Refuse to answer	99	
d3	Do you get help from the government during water logging?	Yes	1	
		No	2	
		Don't know	88	
		Refuse to answer	99	
d4	What type of assistance do you receive from the government during water logging? (Multiple)	Access to clean drinking water	1	
		Temporary shelter	2	
		Food supplies	3	
		Sanitation facilities	4	
		Health services	5	
		Financial support	6	
		I don't get any support	7	
		Other (please specify)	77	
		Don't know	88	
		Refuse to answer	99	
**da**	**Coping Mechanisms and Recommendations**			
d5	What personal or household strategies do you use to cope with water logging? (Multuple)	Storing extra water	1	
		Using water purifiers or boiling water	2	
		Building barriers to prevent water entry	3	
		Relocating to safer areas temporarily	4	
		Other (please specify)	5	
		Don't know	88	
		Refuse to answer	99	
d6	What additional support do you think is necessary from the community to better cope with water logging? (Multiple)	More access to clean drinking water	1	
		Better temporary shelters	2	
		Increased food supplies	3	
		Improved sanitation facilities	4	
		Enhanced health services	5	
		Financial support	6	
		Other (please specify)	77	
		Don't know	88	
		Refuse to answer	99	
d7	What additional support do you think is necessary from the government to better cope with water logging? (Multiple)	More access to clean drinking water	1	
		Better temporary shelters	2	
		Increased food supplies	3	
		Improved sanitation facilities	4	
		Enhanced health services	5	
		Financial support	6	
		Other (please specify):	77	

Original Questionnaire

SL	Questions	Answers (Please tick)	Code	Skip
		Don't know	88	
		Refuse to answer	99	
**C**	**Rapid Observation**			
c1	May we have your permission to see your household water storage, toilet, and handwashing place?	Yes	1	
		No	2	
c1	**WASH observation during non-logging periods**			
**c1w**	**Water observation during non-logging periods**			
c1w1	Can you show me the drinking water source? (select_one)	Piped water-Piped into dwelling	1	
		Piped water-Piped into compound, yard or plot	2	
		Piped water-Piped to neighbour	3	
		Piped water-Public tap/standpipe	4	
		Piped water-Borehole or tubewell	5	
		Dug well-Protected well	6	
		Dug well-Unprotected well	7	
		Water from spring-Protected spring	8	
		Water from spring-Unprotected spring	9	
		Rainwater collection	10	
		Delivered water-Tanker-truck	11	
		Delivered water-Cart with small tank/drum	12	
		Water kiosk	13	
		Packaged water-Bottled water	14	
		Packaged water-Sachet water	15	
		Surface water (river, stream, dam, lake, pond, canal, irrigation channel)	16	
		Can not observe	17	
		Other (specify)	77	
		Not sure	88	
c1w2	Can I see your household's drinking water storage container? (select_one)	Water not store in containers	1	
		Water store in covered containers	2	
		Water store in uncovered containers	3	
		None available	4	
		Can not observe	5	
		Other (Specify):	77	
		Not sure	88	
c1w3	Presence of drinking water treatment facilities (select_multiple)	Boil	1	
		Add bleach/chlorine	2	
		Strain it through a cloth	3	
		Use water filter (ceramic, sand, composite, etc.)	4	

Original Questionnaire

SL	Questions	Answers (Please tick)	Code	Skip
		Solar disinfrction	5	
		Let it stand and settle	6	
		None	7	
		Can not observe	8	
		Other (specify)	77	
		Not sure	88	
**c1s**	**Sanitation Facility Observation during non-logging periods**			
c1s1	Type of Toilet Facilities (select_one)	**Flush/pour flush**-Flush to piped sewer system	1	
		**Flush/pour flush**-Flush to septic tank	2	
		**Flush/pour flush**-Flush to pit latrine	3	
		**Flush/pour flush**-Flush to open drain	4	
		**Flush/pour flush**-Flush to don't know where	5	
		**Dry pit latrines**-Pit latrine with slab	6	
		**Dry pit latrines**-Pit latrine without slab / Open pit	7	
		**Composting toilets-**Twin pit with slab	8	
		**Composting toilets**-Twin pit without slab	9	
		**Composting toilets**-Other composting toilet	10	
		**Bucket**-Container based sanitation	11	
		**Bucket**-Hanging toilet / hanging latrine	12	
		No facility / Bush / Field	13	
		Not sure	88	
c1s2	Observation about access to toilet. (select_multiple)	Path is clear	1	
		Dense vegetation in front of toilet	2	
		Wate or debris on path	3	
		Major crevice or potholes on path	4	
		Mud on path	5	
		Entrance to toilet is obstructed	6	
		None	7	
		Not sure	88	
**c1h**	**Handwashing facility observation during non-logging periods**			
c1h1	Where do you and other member of your household most often wash your hands? (select_one)	Fixed facility reported (sink/tap)-In dwelling	1	
		Fixed facility reported (sink/tap)-In yard/plot	1	
		Mobile object reported (bucket/jug/kettle)	2	

Original Questionnaire

SL	Questions	Answers (Please tick)	Code	Skip
		No handwashing place in dwelling/yard/plot	3	
		Others (Specify)	77	
c1h2	Observe presence of water at the place for handwashing. (select_one)	Water is available	1	
		Water is not available	4	
c1h3	Is soap or detergent present at the place for handwashing? (select_one)	Yes, present	1	
		No, not present	4	
c1h4	If yes (select_multiple)	Soap	1	
		Detergent	2	
		Ash	3	
		Mud/sand	4	
		Others (Specify)	77	
		Not sure	88	
**c2**	**WASH observation during water logging periods**			
**c2w**	**Water observation during water-logging periods**			
c2w1	During the latest water logging period, did you and your family use the same main source of drinking water as when there was non-logging?	Yes	1	
		No	2	
c2w2	Can you show me the drinking water source during water-logging periods? (select_one)	Piped water-Piped into dwelling	1	
		Piped water-Piped into compound, yard or plot	2	
		Piped water-Piped to neighbour	3	
		Piped water-Public tap/standpipe	4	
		Piped water-Borehole or tubewell	5	
		Dug well-Protected well	6	
		Dug well-Unprotected well	7	
		Water from spring-Protected spring	8	
		Water from spring-Unprotected spring	9	
		Rainwater collection	10	
		Delivered water-Tanker-truck	11	
		Delivered water-Cart with small tank/drum	12	
		Water kiosk	13	
		Packaged water-Bottled water	14	
		Packaged water-Sachet water	15	
		Surface water (river, stream, dam, lake, pond, canal, irrigation channel)	16	
		Other (specify)	77	
c2w3	Can I see your household’s drinking water storage	Water not store in containers	1	
		Water store in covered containers	2	
		Water store in uncovered containers	3	

SL	Questions	Answers (Please tick)	Code	Skip
	container during water-logging periods? (select_one)	None available	4	
		Other (Specify):	77	
		Not sure	88	
c2w4	Presence of drinking water treatment facilities (select_one)	Boil	1	
		Add bleach/chlorine	2	
		Strain it through a cloth	3	
		Use water filter (ceramic, sand, composite, etc.)	4	
		Solar disinfrction	5	
		Let it stand and settle	6	
		Other (specify)	77	
		Not sure	88	
**c2s**	**Sanitation Facility (Toilet) Observation during water-logging periods**			
c2s1	Did you and your household uses a different toilet facilities during the latest water-logging period?	Yes	1	
		No	2	
c2s2	Type of Toilet Facilities (select_one)	**Flush/pour flush**-Flush to piped sewer system	1	
		**Flush/pour flush**-Flush to septic tank	2	
		**Flush/pour flush**-Flush to pit latrine	3	
		**Flush/pour flush**-Flush to open drain	4	
		**Flush/pour flush**-Flush to don't know where	5	
		**Dry pit latrines**-Pit latrine with slab	6	
		**Dry pit latrines**-Pit latrine without slab / Open pit	7	
		**Composting toilets-**Twin pit with slab	8	
		**Composting toilets**-Twin pit without slab	9	
		**Composting toilets**-Other composting toilet	10	
		**Bucket**-Container based sanitation	11	
		**Bucket**-Hanging toilet / hanging latrine	12	
		No facility / Bush / Field	13	
		Not sure	88	
c2s3	Type of toilet (select_one)	Private (One household)	1	
		Shared (more than one household)	2	
		Public	3	
		Not sure	88	

Original Questionnaire

SL	Questions	Answers (Please tick)	Code	Skip
c2s4	Observation about access to toilet. (select_multiple)	Path is clear	1	
		Dense vegetation in front of toilet	2	
		Wate or debris on path	3	
		Major crevice or potholes on path	4	
		Mud on path	5	
		Entrance to toilet is obstructed	6	
		None	7	
		Not sure	88	
**c2h**	**Handwashing facility observation during water-logging periods**	**Please show me where you normally wash your hands during water-logging periods**		
c2h1	Did you and your household uses a different toilet facilities during the latest water-logging period?	Yes	1	
		No	2	
c2h2	Where do you and other member of your household most often wash your hands? (select_one)	Fixed facility reported (sink/tap)-In dwelling	1	
		Fixed facility reported (sink/tap)-In yard/plot	1	
		Mobile object reported (bucket/jug/kettle)	2	
		No handwashing place in dwelling/yard/plot	3	
		Others (Specify)	77	
c2h3	Observe presence of water at the place for handwashing. (select_one)	Water is available	1	
		Water is not available	2	
c2h4	Is soap or detergent or ash/mud/sand present at the place for handwashing. (select_one)	Yes, present	1	
		No, not present	4	
c2h5	If yes (multiple)	Soap	1	
		Detergent	2	
		Ash	3	
		Mud/sand	4	
		Others (Specify)	77	
		Not sure	88	
