# Supplementary material for: Differences in access to water, sanitation, and hygiene facilities among residents of Korail Slum, Bangladesh, during normal vs. water-logging situations
Source: PLoS One. 2025 Sep 19;20(9):e0332534. doi: 10.1371/journal.pone.0332534 (PMC12449000; doi:10.1371/journal.pone.0332534)
Supplement: S1 Table — (DOCX) [file pone.0332534.s003.docx]

# **Supplementary Table 1. Participants’ education, income, occupation, and reported household asset ownerships**

| **Characteristic** | **Frequency (%), unless otherwise indicated** |
| --- | --- |
| Education: More than primary education (vs. primary education or less) | 268 (66.3%) |
| Income: More than 20k BDT per month (vs. 20k or lower) | 242 (59.9%) |
| **Asset ownership** |  |
| Electricity | 404 (100%) |
| Solar electricity | 2 (0.5%) |
| Television | 228 (56.4%) |
| Refrigerator | 275 (68.1%) |
| Almira/wardrobe | 259 (64.1%) |
| A sofa set | 47 (11.6%) |
| Table/Chair | 196 (48.5%) |
| Electric fan | 380 (94.1%) |
| Water filter | 60 (14.9%) |
| Washing machine | 1 (0.2%) |
| Water pump | 1 (0.2%) |
| IPS/generator | 0 (0%) |
| Air conditioner | 0 (0%) |
| Car/truck/microbus | 8 (2.0%) |
| Auto bike/CNG/tempo | 0 (0%) |
| Motorcycle/motor scooter | 26 (6.4%) |
| Rickshaw/Van | 6 (1.5%) |
| Bicycle | 55 (13.6%) |
| Smart mobile phone | 358 (88.6%) |
| Normal mobile phone | 330 (81.7%) |
| Computer/Laptop | 42 (10.4%) |

*Retained for Principal Component Analysis
