# Supplementary material for: Differences in access to water, sanitation, and hygiene facilities among residents of Korail Slum, Bangladesh, during normal vs. water-logging situations
Source: PLoS One. 2025 Sep 19;20(9):e0332534. doi: 10.1371/journal.pone.0332534 (PMC12449000; doi:10.1371/journal.pone.0332534)
Supplement: S2 Table — (DOCX) [file pone.0332534.s004.docx]

# **Supplementary Table 2. Principal Component loading of selected characteristics or assets as socioeconomic status indicators**

| **Characteristic** | **Loading for RC1** | **Loading for RC2** |
| --- | --- | --- |
| Education: More than primary education (vs. primary education or less) | 0.34 | 0.32 |
| Income: More than 20k BDT per month (vs. 20k or lower) | 0.59* | 0.09 |
| **Asset ownership** |  |  |
| Television | 0.68* | -0.21 |
| Refrigerator | 0.70* | 0.18 |
| Almira/wardrobe | 0.71* | 0.18 |
| A sofa set | 0.45* | 0.08 |
| Table/Chair | 0.53* | 0.25 |
| Water filter | 0.30 | 0.31 |
| Bicycle | 0.03 | 0.34 |
| Smart mobile phone | 0.33 | 0.58* |
| Normal mobile phone | 0.26 | -0.74* |
| Computer/Laptop | 0.38 | -0.01 |
