# Supplementary material for: Differences in access to water, sanitation, and hygiene facilities among residents of Korail Slum, Bangladesh, during normal vs. water-logging situations
Source: PLoS One. 2025 Sep 19;20(9):e0332534. doi: 10.1371/journal.pone.0332534 (PMC12449000; doi:10.1371/journal.pone.0332534)
Supplement: S3 Table — (DOCX) [file pone.0332534.s005.docx]

# **Supplementary Table 3. Characteristics and asset ownership of participants by socioeconomic tertiles**

| **Characteristic** | **Tertile 1 (poorer) (n= 132)** | **Tertile 2 (medium) (n = 138)** | **Tertile 3 (wealthier) (n = 134)** |
| --- | --- | --- | --- |
| Education: Secondary education or higher (vs. primary education or less) | 62 (47.0%) | 89 (64.6%) | 117 (87.3%) |
| Income: More than 20k BDT per month (vs. 20k or lower) | 37 (28.0%) | 81 (58.7%) | 124 (92.5%) |
| **Asset ownership** |  |  |  |
| Television | 19 (14.4%) | 78 (56.5%) | 131 (97.8%) |
| Refrigerator | 32 (24.2%) | 111 (80.4%) | 132 (98.5%) |
| Almira/wardrobe | 21 (15.9%) | 107 (77.5%) | 131 (97.8%) |
| A sofa set | 1 (0.8%) | 9 (6.5%) | 37 (27.6%) |
| Table/Chair | 26 (19.7%) | 62 (44.9%) | 108 (80.6%) |
| Water filter | 7 (5.3%) | 17 (12.3%) | 36 (26.9%) |
| Bicycle | 16 (12.1%) | 20 (14.5%) | 19 (14.2%) |
| Smart mobile phone | 99 (75.0%) | 128 (92.8%) | 131 (97.8%) |
| Normal mobile phone | 97 (73.5%) | 107 (77.5%) | 126 (94.0%) |
| Computer/Laptop | 2 (1.5%) | 10 (7.2%) | 30 (22.4%) |
