# Supplementary material for: Differences in access to water, sanitation, and hygiene facilities among residents of Korail Slum, Bangladesh, during normal vs. water-logging situations
Source: PLoS One. 2025 Sep 19;20(9):e0332534. doi: 10.1371/journal.pone.0332534 (PMC12449000; doi:10.1371/journal.pone.0332534)
Supplement: S4 Table — (DOCX) [file pone.0332534.s006.docx]

# **Supplementary Table 4. Access to water according to the JMP Service Ladder among participating Korail Slum residents during normal vs. water-logging periods (overall and stratified by socioeconomic tertile)**

| **Normal period** | **Water-logging period** | | | | **p-value^d^** |
| --- | --- | --- | --- | --- | --- |
| **Overall** | **Basic^a^** | **Limited** | **Unimproved** | **Surface Water** |  |
| Basic^a^ (n=382) | 382 (100%) | 0 (0%) | 0 (0%) | 0 (0%) | N/A |
| Limited (n=1) | 0 (0%) | 1 (100%) | 0 (0%) | 0 (0%) |  |
| Unimproved (n=0) | 0 (0%) | 0 (0%) | 0 (0%) | 0 (0%) |  |
| Surface water (n=0) | 0 (0%) | 0 (0%) | 0 (0%) | 0 (0%) |  |
| **Among participants in the first tertile (n=127 households)** |  |  |  |  |  |
| Basic (n=127) | 127 (100%) | 0 (0%) | 0 (0%) | 0 (0%) | N/A |
| Limited (n=0) | 0 (0%) | 0 (0%) | 0 (0%) | 0 (0%) |  |
| Unimproved (n=0) | 0 (0%) | 0 (0%) | 0 (0%) | 0 (0%) |  |
| Surface water (n=0) | 0 (0%) | 0 (0%) | 0 (0%) | 0 (0%) |  |
| **Among participants in the second tertile (n=130 households)** |  |  |  |  |  |
| Basic (n=129) | 129 (100%) | 0 (0%) | 0 (0%) | 0 (0%) | N/A |
| Limited (n=1) | 0 (0%) | 1 (100%) | 0 (0%) | 0 (0%) |  |
| Unimproved (n=0) | 0 (0%) | 0 (0%) | 0 (0%) | 0 (0%) |  |
| Surface water (n=0) | 0 (0%) | 0 (0%) | 0 (0%) | 0 (0%) |  |
| **Among participants in the third tertile (n=126 households)** |  |  |  |  |  |
| Basic (n=126) | 126 (100%) | 0 (0%) | 0 (0%) | 0 (0%) | N/A |
| Limited (n=0) | 0 (0%) | 0 (0%) | 0 (0%) | 0 (0%) |  |
| Unimproved (n=0) | 0 (0%) | 0 (0%) | 0 (0%) | 0 (0%) |  |
| Surface water (n=0) | 0 (0%) | 0 (0%) | 0 (0%) | 0 (0%) |  |
| Breslow-Day Test p-value = N/A |  |  |  |  |  |

^a^May or may not include safely managed due to lack of data on faecal contamination testing;

^b^May or may not include safely managed due to lack of data on disposal of excreta from onsite sanitation facilities

^c^Handwashing behaviors during the “normal period” refers to behaviors within the week prior to the survey; those who answered “Don’t know”, “Refuse to answer”, or “Not applicable” were excluded from the analyses

^d^Based on McNemar’s Test
