# Supplementary material for: Differences in access to water, sanitation, and hygiene facilities among residents of Korail Slum, Bangladesh, during normal vs. water-logging situations
Source: PLoS One. 2025 Sep 19;20(9):e0332534. doi: 10.1371/journal.pone.0332534 (PMC12449000; doi:10.1371/journal.pone.0332534)
Supplement: S5 Table — (DOCX) [file pone.0332534.s007.docx]

# **Supplementary Table 5. Access to sanitation facilities according to the JMP Service Ladder among participating Korail Slum residents during normal vs. water-logging periods (overall and stratified by socioeconomic tertile)**

| **Normal period** | **Water-logging period** | | | | |
| --- | --- | --- | --- | --- | --- |
| **Overall** | **Basic^a^** | **Limited** | **Unimproved** | **Open defecation** | **p-value^b^** |
| Basic^b^ (n=84) | 76 (90.5%) | 8 (9.5%) | 0 (0%) | 0 (0%) | 0.383 |
| Limited (n=299) | 13 (4.3%) | 286 (95.7%) | 0 (0%) | 0 (0%) |  |
| Unimproved (n=0) | 0 (0%) | 0 (0%) | 0 (0%) | 0 (0%) |  |
| Open defecation (n=0) | 0 (0%) | 0 (0%) | 0 (0%) | 0 (0%) |  |
| **Among participants in the first tertile (n=127 households)** |  |  |  |  |  |
| Basic^a^ (n=11) | 10 (90.9%) | 1 (9.1%) | 0 (0%) | 0 (0%) | 0.371 |
| Limited (n=116) | 4 (3.4%) | 112 (96.6%) | 0 (0%) | 0 (0%) |  |
| Unimproved (n=0) | 0 (0%) | 0 (0%) | 0 (0%) | 0 (0%) |  |
| Open defecation (n=0) | 0 (0%) | 0 (0%) | 0 (0%) | 0 (0%) |  |
| **Among participants in the second tertile (n=130 households)** |  |  |  |  |  |
| Basic^a^ (n=27) | 23 (85.2%) | 4 (14.8%) | 0 (0%) | 0 (0%) | 0.999 |
| Limited (n=103) | 3 (2.9%) | 100 (97.1%) | 0 (0%) | 0 (0%) |  |
| Unimproved (n=0) | 0 (0%) | 0 (0%) | 0 (0%) | 0 (0%) |  |
| Open defecation (n=0) | 0 (0%) | 0 (0%) | 0 (0%) | 0 (0%) |  |
| **Among participants in the third tertile (n=126 households)** |  |  |  |  |  |
| Basic^a^ (n=46) | 43 (93.5%) | 3 (6.5%) | 0 (0%) | 0 (0%) | 0.505 |
| Limited (n=80) | 6 (7.5%) | 74 (92.5%) | 0 (0%) | 0 (0%) |  |
| Unimproved (n=0) | 0 (0%) | 0 (0%) | 0 (0%) | 0 (0%) |  |
| Open defecation (n=0) | 0 (0%) | 0 (0%) | 0 (0%) | 0 (0%) |  |
| Breslow-Day Test p-value = 0.944 | | | | | |

^a^May or may not include safely managed due to lack of data on disposal of excreta from onsite sanitation facilities

^b^Based on McNemar’s Test
