# Supplementary material for: Differences in access to water, sanitation, and hygiene facilities among residents of Korail Slum, Bangladesh, during normal vs. water-logging situations
Source: PLoS One. 2025 Sep 19;20(9):e0332534. doi: 10.1371/journal.pone.0332534 (PMC12449000; doi:10.1371/journal.pone.0332534)
Supplement: S6 Table — (DOCX) [file pone.0332534.s008.docx]

# **Supplementary Table 6. Access to hygiene facilities according to the JMP Service Ladder and self-reported frequency of handwashing behaviors among participating Korail Slum residents during normal vs. water-logging periods (overall and stratified by socioeconomic tertile)**

| **During the normal period** | **During water-logging period** | | | |
| --- | --- | --- | --- | --- |
|  | **Basic** | **Limited** | **No facility** | **p-value^a^** |
| **ACCESS TO HYGIENE FACILITIES** |  |  |  |  |
| **Overall (n=382 households)** |  |  |  |  |
| Basic (n=324) | 309 (95.4%) | 12 (3.7%) | 3 (0.9%) | 0.134 |
| Limited (n=50) | 19 (38.0%) | 22 (44.0%) | 9 (18.0%) |  |
| No facility (n=8) | 1 (12.5%) | 3 (37.5%) | 4 (50.0%) |  |
| **Access to hygiene facilities among participants in the first tertile (n=127 households)** |  |  |  |  |
| Basic (n=113) | 107 (94.7%) | 4 (3.5%) | 2 (1.8%) | 0.545 |
| Limited (n=10) | 1 (10.0%) | 8 (80.0%) | 1 (10.0%) |  |
| No facility (n=4) | 1 (25.0%) | 1 (25.0%) | 2 (50.0%) |  |
| **Access to hygiene facilities among participants in the second tertile (n=129 households)** |  |  |  |  |
| Basic (n=117) | 113 (96.6%) | 3 (2.6%) | 1 (0.9%) | NA |
| Limited (n=12) | 6 (50.0%) | 3 (25.0%) | 3 (25.0%) |  |
| No facility (n=0) | 0 (0%) | 0 (0%) | 0 (0%) |  |
| **Access to hygiene facilities among participants in the third tertile (n=126 households)** |  |  |  |  |
| Basic (n=94) | 89 (94.7%) | 5 (5.3%) | 0 (0%) | NA |
| Limited (n=28) | 12 (42.9%) | 11 (39.3%) | 5 (17.9%) |  |
| No facility (n=4) | 0 (0%) | 2 (50.0%) | 2 (50.0%) |  |
| **SELF-REPORTED FREQUENCY OF HANDWASHING BEHAVIORS** |  |  |  |  |
| **HANDWASHING WITH WATER AND SOAP AFTER GOING TO THE TOILET** |  |  |  |  |
| **Overall (n=383 households)** | **Not always** | **Always** |  |  |
| Not always (n=324) | 318 (98.1%) | 6 (1.9%) |  | 0.117 |
| Always (n=59) | 14 (23.7%) | 45 (76.3%) |  |  |
| **Among participants in the first tertile (n=127 households)** | **Not always** | **Always** |  |  |
| Not always (n=116) | 113 (97.4%) | 3 (2.6%) |  | 0.999 |
| Always (n=11) | 2 (18.2%) | 9 (81.8%) |  |  |
| **Among participants in the second tertile (n=130 households)** | **Not always** | **Always** |  |  |
| Not always (n=106) | 106 (100%) | 0 (0.0%) |  | 0.023 |
| Always (n=24) | 7 (29.2%) | 17 (70.8%) |  |  |
| **Among participants in the third tertile (n=126 households)** | **Not always** | **Always** |  |  |
| Not always (n=102) | 99 (97.1%) | 3 (2.9%) |  | 0.724 |
| Always (n=24) | 5 (20.8%) | 19 (79.2%) |  |  |
| **HANDWASHING WITH WATER AND SOAP AFTER CHANGING A CHILD’S SOILED DIAPERS** |  |  |  |  |
| **Overall (n=179 households)** | **Not always** | **Always** |  |  |
| Not always (n=141) | 138 (97.8%) | 3 (2.2%) |  | 0.505 |
| Always (n=38) | 6 (15.8%) | 32 (84.2%) |  |  |
| **Among participants in the first tertile (n=45 households)** | **Not always** | **Always** |  |  |
| Not always (n=37) | 37 (100%) | 0 (0%) |  | 0.480 |
| Always (n=8) | 2 (25.0%) | 6 (75.0%) |  |  |
| **Among participants in the second tertile (n=73 households)** | **Not always** | **Always** |  |  |
| Not always (n=58) | 56 (96.6%) | 2 (3.4%) |  | 0.999 |
| Always (n=15) | 1 (6.7%) | 14 (93.3%) |  |  |
| **Among participants in the third tertile (n=61 households)** | **Not always** | **Always** |  |  |
| Not always (n=46) | 45 (97.8%) | 1 (2.2%) |  | 0.617 |
| Always (n=15) | 3 (20.0%) | 12 (80.0%) |  |  |
| **HANDWASHING WITH WATER AND SOAP BEFORE PREPARING FOOD** |  |  |  |  |
| **Overall (n=344 households)** |  |  |  |  |
| Not always (n=314) | 311 (99.0%) | 3 (1.0%) |  |  |
| Always (n=30) | 9 (30.0%) | 21 (70.0%) |  |  |
| **Among participants in the first tertile (n=108 households)** | **Not always** | **Always** |  |  |
| Not always (n=101) | 101 (100%) | 0 (0%) |  | 0.480 |
| Always (n=7) | 2 (28.6%) | 5 (71.4%) |  |  |
| **Among participants in the second tertile (n=122 households)** | **Not always** | **Always** |  |  |
| Not always (n=110) | 109 (99.1%) | 1 (0.9%) |  | 0.371 |
| Always (n=12) | 4 (33.3%) | 8 (66.7%) |  |  |
| **Among participants in the third tertile (n=114 households)** | **Not always** | **Always** |  |  |
| Not always (n=103) | 101 (98.1%) | 2 (1.9%) |  | 0.999 |
| Always (n=11) | 3 (27.3%) | 8 (72.7%) |  |  |
| **HANDWASHING WITH WATER AND SOAP BEFORE FEEDING A CHILD** |  |  |  |  |
| **Overall (n=184 households)** | **Not always** | **Always** |  |  |
| Not always (n=155) | 155 (100%) | 0 (0%) |  | 0.074 |
| Always (n=29) | 5 (17.2%) | 24 (82.8%) |  |  |
| **Among participants in the first tertile (n=47 households)** | **Not always** | **Always** |  |  |
| Not always (n=43) | 43 (100%) | 0 (0%) |  | NA |
| Always (n=4) | 0 (0%) | 4 (100%) |  |  |
| **Among participants in the second tertile (n=72 households)** | **Not always** | **Always** |  |  |
| Not always (n=58) | 58 (100%) | 0 (0%) |  | 0.248 |
| Always (n=14) | 3 (21.4%) | 11 (78.6%) |  |  |
| **Among participants in the third tertile (n=65 households)** | **Not always** | **Always** |  |  |
| Not always (n=54) | 54 (100%) | 0 (0%) |  | 0.479 |
| Always (n=11) | 2 (18.2%) | 9 (81.8%) |  |  |
| **HANDWASHING WITH WATER AND SOAP BEFORE EATING** |  |  |  |  |
| **Overall (n=364 households)** | **Not always** | **Always** |  |  |
| Not always (n=343) | 340 (99.1%) | 3 (0.9%) |  | 0.343 |
| Always (n=31) | 7 (22.6%) | 24 (77.4%) |  |  |
| **Among participants in the first tertile (n=124 households)** | **Not always** | **Always** |  |  |
| Not always (n=118) | 117 (99.2%) | 1 (0.8%) |  | 0.999 |
| Always (n=6) | 0 (0%) | 6 (100%) |  |  |
| **Among participants in the second tertile (n=126 households)** | **Not always** | **Always** |  |  |
| Not always (n=113) | 111 (98.2%) | 2 (1.8%) |  | 0.683 |
| Always (n=13) | 4 (30.8%) | 9 (69.2%) |  |  |
| **Among participants in the third tertile (n=124 households)** | **Not always** | **Always** |  |  |
| Not always (n=112) | 112 (100%) | 0 (0%) |  | 0.248 |
| Always (n=12) | 3 (25%) | 9 (75%) |  |  |

^a^Based on McNemar’s Test
