# Supplementary material for: Differences in access to water, sanitation, and hygiene facilities among residents of Korail Slum, Bangladesh, during normal vs. water-logging situations
Source: PLoS One. 2025 Sep 19;20(9):e0332534. doi: 10.1371/journal.pone.0332534 (PMC12449000; doi:10.1371/journal.pone.0332534)
Supplement: Attachment — Submitted filename: R1 - Response to Reviewers-20250704.docx [file pone.0332534.s010.docx]

**Response to Reviewers**

| **COMMENT** | **RESPONSE** |
| --- | --- |
| **Editor’s Comment / Journal Requirements** |  |
| COMMENT  1. Please ensure that your manuscript meets PLOS ONE's style requirements, including those for file naming. The PLOS ONE style templates can be found at  https://journals.plos.org/plosone/s/file?id=wjVg/ PLOSOne_formatting_sample_main_body.pdf and  https://journals.plos.org/plosone/s/file?id=ba62/ PLOSOne_formatting_sample_title_authors_affiliations.pdf | RESPONSE  We thank the editor for the comment. We have revised the manuscript accordingly. |
| COMMENT  2. In the ethics statement in the Methods, you have specified that verbal consent was obtained. Please provide additional details regarding how this consent was documented and witnessed, and state whether this was approved by the IRB | RESPONSE  Thank you for this important comment. We have revised the Ethics section in the Methods to clarify the procedures for verbal consent. Specifically, we have added the following information:  “We requested and received ethical approval for this study from the Bangladesh Medical Research Council (BMRC) (Ref: BMRC/NREC/2022-2025/561). Due to the low literacy levels among the residents of Korail Slum, we requested and received approval for a waiver of written informed consent and the use of only verbal informed consent.  We obtained verbal informed consent from all participants before initiating data collection. This process included reading a consent script aloud. The consent script included a summary of the purpose of the study, procedures, risks, benefits, and confidentiality. We gave the participants an opportunity to ask questions before verbally agreeing to participate or declining participation. Our research assistants documented the verbal consent directly in the electronic questionnaire form on KoboCollect. The form required an affirmative response regarding verbal consent to display the data collection questionnaire.” |
| COMMENT  3. Thank you for stating the following in the Acknowledgments Section of your manuscript:  “The first author (MMR) received financial support for data collection in this research work from the TUYF Charitable Trust: Research Capacity through Education and Networking on Epidemiology in Asia, the Department of Epidemiology, Faculty of Medicine, Prince of Songkla University (Grant number 1/2023). The funders had no role in study design, data collection and analysis, decision to publish, or preparation of the manuscript.”  We note that you have provided funding information that is not currently declared in your Funding Statement. However, funding information should not appear in the Acknowledgments section or other areas of your manuscript. We will only publish funding information present in the Funding Statement section of the online submission form.  Please remove any funding-related text from the manuscript and let us know how you would like to update your Funding Statement. Currently, your Funding Statement reads as follows:  “The first author (MMR) received financial support for data collection in this research work from the TUYF Charitable Trust: Research Capacity through Education and Networking on Epidemiology in Asia, the Department of Epidemiology, Faculty of Medicine, Prince of Songkla University (Grant number 1/2023). The funders had no role in study design, data collection and analysis, decision to publish, or preparation of the manuscript.”  Please include your amended statements within your cover letter; we will change the online submission form on your behalf. | RESPONSE  Thank you for your guidance. We have removed the funding-related text from the manuscript. We wish to maintain the same funding statement as it appears, but we will leave the decision on where to place the statement at the editors’ discretion. We have included the following remarks in the cover letter for this revision.    “We wish to take this opportunity to request the Journal to state the following remarks in the Funding Statement, should the manuscript be accepted for publication by PLoS ONE:  “The first author (MMR) received financial support for data collection in this research work from the TUYF Charitable Trust: Research Capacity through Education and Networking on Epidemiology in Asia, the Department of Epidemiology, Faculty of Medicine, Prince of Songkla University (Grant number 1/2023). The funders had no role in study design, data collection and analysis, decision to publish, or preparation of the manuscript.” |
| COMMENT  4. Please include captions for your Supporting Information files at the end of your manuscript, and update any in-text citations to match accordingly. Please see our Supporting Information guidelines for more information: http://journals.plos.org/plosone/s/supporting-information. | RESPONSE  We thank the Editor for the comments. We have included captions for the Supporting Information files at the end of the manuscript accordingly. |
| **Reviewers' comments:** |  |
| **Reviewer #1:** |  |
| COMMENT  Dear Author,  This paper examines the impact of waterlogging on slum residents’ handwashing behaviours and access to WASH facilities in Dhaka. However, several areas require adjustment to meet publishing standards, as detailed below: | RESPONSE  Thank you for your valuable comments and suggestions to improve the clarity, quality, and scientific rigor of our manuscript. We have tried to revise the manuscript and address each point raised to the extent possible. |
| COMMENT  1. Language and Style: The language used in this paper requires proofreading and polishing to adhere to academic writing standards. The current manuscript contains several grammatical and structural errors that hinder the clear and complete expression of the research findings and their implications. | RESPONSE  We appreciate this feedback. We have thoroughly proofread and revised the entire manuscript for language, grammar, and clarity to ensure adherence to academic writing standards. |
| COMMENT  2. Introduction: On Introduction page two, lines 1-3, the study focuses on slum residents, while the term 'wealth' suggests a broader scope. Please clarify why data collected from slum residents can be used to analyse 'disparities in these differences by wealth' and provide more detail on how this data contributes to the analysis. | RESPONSE  Thank you for pointing this out. We just want to clarify that we did not select slum residents specifically for the study of socioeconomic disparities. We measured WASH access among slum residents due to their relative vulnerability (particularly during water logging periods compared to normal periods), and we needed to measure the extent to which socioeconomic disparities occurred in this context. The idea behind this assessment was that even among those who lived in the slums, there was heterogeneity regarding socioeconomic conditions. In various surveys (such as the MICS) asset ownership is used as a proxy indicator of socioeconomic status. We used the same approach in this study. We have revised the INTRODUCTION section as follows:  “Although slum residents are generally less wealthy than residents of other parts of a city, heterogeneity in socioeconomic conditions (i.e., wealth disparities) can also be found within a slum and can be measured using a combination of socioeconomic characteristics and asset ownership. Despite concerns regarding the vulnerability of slum residents to climate events and the general notion that climate events affect the poor more commonly and to a greater extent compared to the rich, studies have not described differences in access to water, sanitation, and hygiene (WASH) facilities within a household between normal and water-logging periods among slum residents. Studies also have not described the disparities of these differences by wealth. ” |
| COMMENT  3. Methods: On Methods page one, line 7, please provide a source for the population data. | RESPONSE  We thank the reviewer for the comment. We have added details to the Sampling Method sub-section with citations accordingly. |
| COMMENT  4. Methods: On Methods page one, line 9, please include a figure to illustrate the location and spatial characteristics of the slum. | RESPONSE  We thank the reviewer for the suggestion. We have added Figure 1, which includes a map of Korail Slum with the location in Dhaka and proximity to key city landmarks. |
| COMMENT  5. Sampling Method: On the sampling method page, line 7, please provide further details on the origin of the number '16536,' as this is the first instance of its appearance. | RESPONSE  We thank the reviewer for the comment. We have added details to the Sampling Method sub-section with citations accordingly. |
| COMMENT  6. Study Instruments: On the study instruments page, line 15, the term 'smartphone-based face-to-face structured interview questionnaire' may benefit from rephrasing for improved clarity. | RESPONSE  We have revised the remarks to the following to improve clarity:  “Our study instrument was a structured questionnaire hosted on the web-based KoboToolbox platform. We used smartphones with either a web browser or the KoboCollect application for data collection.” |
| COMMENT  7. Study Instruments: On the study instruments page, line 18, it would be beneficial to include citations for the 'previous studies' mentioned. | RESPONSE  We thank the reviewer for the suggestion. We have added the citations accordingly. |
| COMMENT  8. Study Variables: On the study variables page, lines 3-5, 'waterlogging' is presented as a general term representing varying degrees of reduced access to WASH facilities due to surface water. Please specify the quality control measures employed to ensure consistency in how interviewees perceived and reported 'waterlogging.' | RESPONSE  We thank the reviewer for the comment. We have added the following remarks to the Study Variables sub-section in METHODS:  "Our operational definition of the term "water-logging" was "inundation of least 25 cm in depth lasting 10 hours or more." We trained our research assistants to explain this definition in a uniform manner to all participants when conducting interviews about experiences during water-logging periods." |
| COMMENT  9. Study Variables: On the study variables page, line 21, provide more detailed information about 'the most recent water-logging period,' including its scale and severity (e.g., water depth). This will clarify whether all interviewees referred to the same event or events with comparable impacts. | RESPONSE  We thank the reviewer for the comment. We have added the following remarks to the Study Variables sub-section in METHODS:  "We asked participants to describe their level of access to water, sanitation, and hygiene during the most recent water-logging periods within the past 12 months that the participants experienced without giving the specific dates." |
| COMMENT  10. Effect Modifier Variable: On the effect modifier variable page, line 11, please elaborate on the 'loading scores' method used to categorize participants into tertiles. | RESPONSE  We thank the reviewer for the suggestion. We made a mistake in writing. We did not use the loading to rank the participants, we used the PCA scores. We apologize for our mistake and have made corrections in the manuscript texts accordingly as follows:  “The investigators then followed the instructions for Principal Component Analysis (PCA) and obtained the factor scores. We then performed descriptive statistical analysis on the factor scores and used the 33rd and 67th percentiles as the cut-off points for the socioeconomic tertiles.” |
| COMMENT  11. Results: On the results page, line 8, please provide context for the income figure of 20,000 Taka per household per month by indicating its relative level within the Bangladeshi income distribution. | RESPONSE  We thank the reviewer for the comment. Although the exact percentile could not be determined based on existing data, the household income of 20,000 BDT per month is approximately 45 percent of the average income in urban areas and 77 percent of the average income in rural areas in 2022 according to the Bureau of Statistics  Citation: https://bbs.portal.gov.bd/sites/default/files/files/bbs.portal.gov.bd/page/57def76a_aa3c_46e3_9f80_53732eb94a83/2023-04-13-09-35-ee41d2a35dcc47a94a595c88328458f4.pdf    We feel that based on the need to mention statistics, the addition of the context would be more appropriate elsewhere. Thus, we added the following remarks in the METHODS section.  ""The household income of 20,000 BDT per month was approximately half of the average household income in urban areas of Bangladesh in 2022, and we used the amount as a proxy cut-off point for being low-income in the slum setting."" |
| COMMENT  12. Discussion: On the discussion page, line 20, if the reference pertains to open defecation during 'flooding,' clarify the relationship between 'waterlogging' and 'flooding' in the definition of waterlogging (i.e., whether waterlogging represents minor flooding, and how ‘minor’ it is). | RESPONSE  Thank you for your comment. We have re-checked the reference document, and revised the remark in the DISCUSSION section from the following:  "This contrasted starkly with findings from 2000 to 2010 that open defecation was common in urban slums, particularly during flooding [Reference]. "    To the following:  "The absence of open defecation suggested that the prevalence of open defecation in Bangladesh continued to decline after the reduction to one percent in 2015[Reference]" |
| COMMENT  13. Discussion: The discussion section would benefit from addressing some of the interesting results presented. For example, Table 2 shows that 299 households had access to limited sanitation facilities under normal conditions, but 13 of these households reported access to basic sanitation facilities during waterlogging. Please provide a discussion of this finding. | RESPONSE  We appreciate the reviewer’s suggestion. We have added the following remarks in the DISCUSSION section:  "The majority of households had limited sanitation during normal periods due to the act of sharing their improved latrine with people from other households. However, a small minority (approximately four percent) of participants from these households reported having access to basic (non-shared) sanitation facilities during water-logging periods. We do not know what accounted for this shift, although a previous study reported that slum residents would relocate housing within the same area during water-logging, such as with family members, and relocated residence might have non-shared toilet. Future studies should consider asking questions regarding temporary relocation during water-logging periods." |
| COMMENT  14. Discussion: Consider adding a discussion on the potential resilience of basic and limited WASH facilities to waterlogging, and how this might offer an alternative interpretation of the results. | RESPONSE  We thank the reviewer for the comment. We actually considered the more dynamic shifts in hygiene access and included the following remarks in the DISCUSSION section:  "With regard to access to hygiene facilities, households with basic hygiene during normal periods showed robustness during water-logging periods, whereas some households with limited or no facilities during normal periods had interestingly higher levels of access during water-logging periods. The upward shift could be attributed in part to either relocation or distribution of soap for hand hygiene by civil societies during events. On a related note, hand hygiene behaviors may need improvement." |
| COMMENT  15. Results and Discussion: In addition to the reported frequencies of handwashing during normal and waterlogging periods, it would be valuable to include the frequencies of access to WASH facilities during these periods. For instance, while 76 out of 84 households with access to basic sanitation facilities in normal periods retained access during waterlogging, please discuss whether their usage frequency was affected. The questionnaire appears to contain relevant and valuable data and expanding the discussion of this information would enhance the paper's research significance. | RESPONSE  We thank the reviewer for the comment. Unfortunately, we did not ask the participants about behaviors pertaining to water and sanitation (e.g., the frequency of drawing water or using sanitation facilities). We have added the following remarks to the DISCUSSION section regarding this issue:  "One additional issue in our data is that we did not collect information regarding water and sanitation related behaviors (e.g., the frequency of drawing water or using sanitation facilities). Future studies should consider including behavioral information in order to understand water and sanitation access in a more complete manner." |
| COMMENT  16. Presentation: In Tables 2-5, consider improving the presentation of the data. For example, in Table 2, ensure consistency in the formatting (e.g., font size, background colour) of 'Basic' in both the vertical and horizontal axes. | RESPONSE  We thank the reviewer for the comment. As we are not sure how the manuscript would look during production, an the best way to format the tables to present in such layouts, we wish to refer to the journal's formatting staff for additional suggestions in the production phase of the publication process. |
| COMMENT  17. Table 5: Table 5 requires further clarification. For instance, for the 116 households reported as not always having access to hand hygiene facilities during normal periods, please specify whether this refers to access to basic or limited facilities. | RESPONSE  Thank you for the comment. We believe that the lack of clarity comes from the use of repeated header of the table. The table header was meant for the first part (i.e., access to hand hygiene facilities) whereas the “always” vs. “not always” categorization refers to the second part (i.e., self-reported hand hygiene behavior), for which the table header for the first part was completely irrelevant. We have modified the structure of Table 2 and Table 5 accordingly. |
| COMMENT  Addressing these points will significantly strengthen the manuscript. | RESPONSE  Thank you for the encouraging remark. We appreciate your constructive feedback and agree that addressing these points has helped improve the clarity, rigor, and overall quality of the manuscript. |
| **REVIEWER #2** |  |
| COMMENT  Reviewer Comments on Manuscript PONE-D-25-21002  This manuscript examines differences in access to water, sanitation, and hygiene (WASH) services among residents of Korail Slum, Dhaka, Bangladesh during normal and water-logged conditions. The topic is important and underrepresented in current literature, particularly in relation to urban slums in South Asia, and the authors have access to a valuable dataset. However, in its current form, the paper requires substantial revisions to reach publishable quality. | RESPONSE We sincerely thank you and the reviewers for your thoughtful and constructive feedback. We have revised the manuscript extensively in response to your comments. Please find our detailed responses below: |
| **Major Concerns:** |  |
| The manuscript is a lack of contextualisation for an international audience. Key concepts such as “water-logging” are not clearly defined or situated within the broader literature. The structure is also problematic, with a particularly weak discussion section that does not clearly address the stated objectives or elaborate on the implications of the findings. Additionally, there are no visual representations of data, which limits reader to understand the geographical and hydrological context of the study. The results section is brief and lacks depth, while the interpretation remains superficial. The paper also fails to engage with relevant recent literature on WASH infrastructure, urban flood resilience, and disaster-related behaviour change. These shortcomings collectively weaken the paper’s potential to contribute meaningfully to global scholarship. | We thank the reviewer for the comment. We have tried to revise the manuscript according to the reviewers’ suggestions accordingly. |
| **Specific Comments:** |  |
| COMMENT  • Currently it lack international context. International readers would benefit from a clearer explanation of the urban flooding phenomenon, how it manifests in Korail Slum, and its relation to broader global challenges in slum-based WASH resilience. | RESPONSE  We thank the reviewer for the comment. We have revised the INTRODUCTION section accordingly, particularly by describing urban flooding in Bangladesh and its impact on WASH and health. |
| COMMENT  • Clarify or remove the discussion of “paddy flooding” in the introduction—it is not currently connected to the context. | RESPONSE  We have removed the “paddy flooding” reference and revised the first paragraph accordingly. |
| COMMENT  • Provide a clear rationale for choosing Korail Slum, emphasizing its unique environmental and demographic challenges. | RESPONSE  We thank the reviewer for the comment and have added the following remarks to the Study Design and Setting sub-section of the METHODS section:    "We selected the Korail Slum due to the geographic location in the middle of Dhaka which eases travel and access, the large population, and the vulnerability of the slum to water-logging." |
| COMMENT  • Add a detailed map of the study area. | RESPONSE  We thank the reviewer for the suggestion. We have added Figure 1, which included a map of Korail Slum with the location in Dhaka and proximity to key city landmarks. |
| COMMENT  • The manuscript includes a large number of tables, but the overall data visualisation could be improved. Consider summarising key findings graphically (e.g. through charts or graphs) to enhance clarity and reader engagement. The statistics provided in the tables can go to the supplementary. | RESPONSE  We thank the reviewer for the comment, and we note and appreciate the call for visualization. The authors have deliberated and decided that as tables could present both frequency and percentage that enable replication of the study findings with greater ease, we wish to retain the presentation of the findings in tables format as they appear. |
| COMMENT  • The discussion section needs clearer subheadings and should directly address the aim and objectives with interpretation. | RESPONSE  We thank the reviewer for the suggestion. As we have made substantial revisions to the DISCUSSION section, and we are not quite sure what sub-headings would be appropriate, we wish to keep the DISCUSSION section as it appears. However, we also remain open to suggestions from the journal’s Editor regarding this matter. |
| COMMENT  • In discussion, clarify the logic behind behaviour regression—for example, why those who report handwashing during normal periods did not maintain the behaviour during water-logging. | RESPONSE  We thank the reviewer for the comment. We have added the following remarks to the DISCUSSION section:  "There was little upward shift and substantial downward shift (i.e., regression) in hand hygiene behavior during normal vs. water logging periods. The reason for the shift is unclear, but it is likely that household members may have felt the need to conserve water for other purposes during water-logging periods, hence the lower prevalence." |
| COMMENT  • Address the unexplained trend where the middle socioeconomic tertile shows the greatest shift in hygiene behaviour. | RESPONSE  We thank the reviewer for the comment. We have added the following remarks to the DISCUSSION section:  "We do not have a clear explanation or theory regarding this pattern of variation. However, the prevalence of reporting regular handwashing with water and soap at both normal and water-logging periods was very low, thus we could not rule out chance as the best explanation for the observed heterogeneity." |
| COMMENT  • Polish the language throughout the manuscript to improve readability and clarity. | RESPONSE  We thank the reviewer for the suggestion and we have made our best attempt to revise the language throughout the manuscript accordingly. |
