# Supplementary material for: Differences in access to water, sanitation, and hygiene facilities among residents of Korail Slum, Bangladesh, during normal vs. water-logging situations
Source: PLoS One. 2025 Sep 19;20(9):e0332534. doi: 10.1371/journal.pone.0332534 (PMC12449000; doi:10.1371/journal.pone.0332534)
Supplement: Attachment — Submitted filename: R2 - Response to Reviewers-20250824.docx [file pone.0332534.s011.docx]

**Response to Reviewers Comments**

**Reviewer #1: Reviewer Comments**

**COMMENT**

Thank you for resubmitting your work and for incorporating the previous feedback. However, I still saw several areas that could benefit from further improvement:
Introduction, line 50, ‘floods are very common…’: please rephrase so readers can get more information about the floods in Bangladesh, i.e. frequency, severity, damage, etc.

**Response**

Thank you for the suggestion. We have revised the sentence to read:

“Bangladesh experiences an average of two to five major floods per year, which can inundate up to two-thirds of the country and cause severe damage to infrastructure, agriculture, and public health systems.”

--------------------------------------------------------------------

**COMMENT**

Introduction, line 75, ‘water, sanitation, and hygiene (WASH): please use WASH from here as you already mentioned the full term in the previous text.

**Response**

Thank you. We have replaced “water, sanitation, and hygiene (WASH)” with simply “WASH” throughout the remainder of the manuscript.

--------------------------------------------------------------------

**COMMENT**

Introduction, line 77, ‘such data are…’: please highlight your research significance and be more specific - what benefits of knowing the disparities of these differences by wealth?

**Response:** We have revised the sentence to clarify the significance as follows:

“Such data can inform targeted interventions by helping public health and urban planning stakeholders identify population groups that are vulnerable to WASH disruptions during climate-related events.”

--------------------------------------------------------------------

**COMMENT**

Methods, line 89, ‘Figure 1’: It's good to see that you've added a figure here, but could you replace it with figures from a different source, such as government documents or other literature? I personally am not inclined to use a screenshot from Google satellite in my paper, but this is just a personal preference rather than a comment.

**Response:** Thank you for the suggestion. The authors have deliberated and decided to keep the figure as it appears. We have made some changes as per the suggestions of another reviewer

--------------------------------------------------------------------

**COMMENT**

Methods, line 90, ‘151,500 people’: please add references here.

**Response:** We thank the reviewer for the comment. We have added one reference accordingly.

--------------------------------------------------------------------

**COMMENT**

Methods, line 97, ‘we excluded…’: more details needed here on how you excluded these potential participants - did you ask their health conditions, or based on the observations only?

**Response:** Thank you for your question. We have revised the sentence to clarify the procedure:

“We excluded individuals who self-reported illnesses or other impairments that they deemed would prevent them from participating fully in the interview.”

--------------------------------------------------------------------

**COMMENT**

Methods, line 126, ‘the lead author…’: please indicate who compared and checked the discrepancies? The lead author or 'we'?

**Response:** We have revised the sentence to the following:

“The lead author (MR) compared the original and the back-translated English versions, identified discrepancies, and made corrections to the corresponding parts of the Bengali translation.”

--------------------------------------------------------------------

**COMMENT**

Methods, line 147, ‘surface water’: lower case ‘s’

**Response:** Thank you for noticing. We have corrected it to “surface water,”

--------------------------------------------------------------------

**COMMENT**

Methods, line 156, ‘captured information…’: please indicate if these data were analysed or included in this paper

**Response:** We have revised the sentence to:

“If a participant switched to a different source during water logging, we would ask the participant to identify the source using the same answer choices as our measurement of access to water during normal periods. We also asked the participants about changes in collection time, water shortages, and treatment practices.”

--------------------------------------------------------------------

**COMMENT**

Methods, line 180, ‘key moments…’: please indicate what 'key moments' are. More information needed here.

**Response:** We have added the following clarification:

“Key moments for hand hygiene referred to events identified by the WHO/UNICEF Joint Monitoring Programme[23] as moments where hand hygiene is essential to prevent disease transmission, namely: after going to the toilet, after changing a child's soiled diaper, before preparing food, before feeding a child, and before eating.”

--------------------------------------------------------------------

**COMMENT**

Methods, line 198, ‘(PCA)’: Move (PCA) before the reference, maybe?

**Response:** We agree with the suggestion and have revised the sentence accordingly so that (PCA) appears before the reference citation.

--------------------------------------------------------------------

**COMMENT**

Methods, line 223, ‘data-related issues’: please indicate what data-related issues are.

**Response:** We have added the following remarks after the term “data-related issues”:

“(e.g., missing values, duplicated entries)”

--------------------------------------------------------------------

**COMMENT**

Results, line 275, ‘however, the difference.’: you may want to rephrase this sentence as you already used 'However' in the last sentence.

**Response:** We thank the reviewer for the comment. The RESULTS section has been extensively revised. We decided to restructure the results tables and move them to the supplementary section (as per the suggestions of another reviewer), and added figures.

--------------------------------------------------------------------

**COMMENT**

Discussion, line 327, ‘as residents shifted…’: More details needed here - was this derived from the data collected? If so, it is worth referring to it.

**Response:** We thank the reviewer for the comment. We decided that the remark was not needed to convey the main idea in the paragraph and decided to delete the sentence.

--------------------------------------------------------------------

**COMMENT**

Discussion, line 329, ‘open defecation in…’: Here you used 'contradicted', could you be more specific on this argument here - in reference 28, people chose open defecation because they were not willing to use a shared pit latrine? Or because there was no pit latrine available at all? Are you making an argument that residents in Korail slum were still willing to share toilets with other people, which is different from the Uganda case?

**Response:** We thank the reviewer for the comment. We have revised the remarks as follow:

“Our findings differed from previous studies, which found that slum residents resorted to open defecation during water logging as latrines became inaccessible[29], and that low socioeconomic status (SES) correlates with reduced resilience of the sanitation infrastructures[3].”

--------------------------------------------------------------------

**COMMENT**

Discussion, line 330, ‘corelates with…’: could you be more specific on 'reduced resilience of the sanitation infrastructures' as the original source was mainly about the diarrhoea cases.

**Response:** Thank you for the comment. We have removed the citation and made the following changes to the remark:

“Our findings differed from previous studies, which found that slum residents resorted to open defecation during water logging as latrines became inaccessible[29], and that low socioeconomic status (SES) correlates with reduced resilience of the sanitation infrastructures[3].”

--------------------------------------------------------------------

**COMMENT**

Discussion, line 359, ‘a previous study…’: More details needed here - what did this previous study find?

**Response:** We thank the reviewer for the comment. We have deleted the sentence in its entirety.

--------------------------------------------------------------------

**COMMENT**

Table 4, line 555: Should the title be 'access to sanitation'?

**Response:** We thank the reviewer for the comment, and we agree. The title has been changed to “JMP Service ladder access to sanitation facilities” accordingly. However, as the other reviewer suggest that we use graphs instead of tables, the current Tables 2 thru Table 5 have been moved to the supplementary information section as Supplementary Tables 4 thru 6. We nonetheless thank the reviewer for the helpful suggestion and for the attention to the presentation of our study findings.

--------------------------------------------------------------------

**COMMENT**

Table 4, line 558: superscript 'b' was not in Table 4

**Response:** We appreciate the careful review. We have revised Table 4 and added the missing superscript ‘b’ where appropriate.

**COMMENT**

Table 5, should ‘0.023’ be non-bold?

**Response:** Yes, we have corrected the formatting and removed the bold typeface from Table 5.

--------------------------------------------------------------------

**COMMENT**

I strongly suggest giving your manuscript another thorough proofreading, as there are still some unresolved structural and grammatical issues I haven't detailed.

**Response:** We sincerely thank the reviewer for this recommendation. We have carefully proofread the manuscript again and addressed all remaining grammatical and structural inconsistencies to improve clarity and readability.

###################################################

\#### End of Comments from Reviewer #1 ####

###################################################

**Reviewer #2: Reviewer Comments**

**COMMENT**

The revised manuscript presents meaningful improvements over the original submission and demonstrates the authors’ effort to address many of the concerns raised in the initial review. The authors revised the manuscript's structure, clarified terminology (such as “water-logging”), and improved contextual framing for a broader audience. The addition of a map (Figure 1) is an enhancement and helps to visualise the study area geographically.
The manuscript now includes clearer subheadings and demonstrates better alignment between objectives, results, and discussion. The clarity have improved meaningfully. However, several areas would benefit from additional refinement before publication:

**Response:** We sincerely thank the reviewer for acknowledging the improvements in our revised manuscript. We appreciate the continued constructive feedback and have further revised the manuscript to enhance clarity, structure, and presentation of findings, as detailed below.

--------------------------------------------------------------------

**COMMENT**

Data Visualization: I am not agreeing with the authors here, while the number of tables remains high, no figures summarising key trends or regression results. The rational about reproducibility around table is not valid as there is no survey data given that one can use to reproduce the tables.

Even simple bar charts or summary visuals would help general readers to interpret findings. Consider moving some tables to supplementary material and adding at least few summary figures.

For example, Tables 2 and 5 could be combine into a single panel figure (e.g., side-by-side bar plots showing access and behaviour across SES groups under normal vs. waterlogged conditions).
Also, authors may use difference plots (e.g., percent change between normal and waterlogged periods by SES) to visually represent which groups were most impacted.
The current satellite map of the study area (Korail Basti) lacks sufficient annotation to be informative and accessible to an international audience unfamiliar with the local geography. Including an inset map for geographic context, along with clearly marked survey locations, would significantly enhance the clarity and interpretability of the figure.

**RESPONSE:**

We thank the reviewer for the comment. We have revised the map in Figure 1 accordingly

With regard to the presentation of the study findings, the authors deliberated and decided that the alluvial plot would be the best way to visualize our study findings. We have modified the contents of the Tables 2 thru 5 and moved them to the Supplementary Information section (as Supplementary Tables 4 thru 6). We have also added Figures 2 thru 4 (alluvial plots) to the RESULTS section accordingly. We have also extensively revised the RESULTS section to suit the restructuring of the results tables and the figures.

--------------------------------------------------------------------

**COMMENT**

Discussion: The discussion could interpret and contextualize more clearly under the appropriate subheadings addressing the aim and objectives of the paper. These might be like
o Changes in WASH access and behaviours during water-logging
o Socioeconomic disparities in WASH access during climate stress
o Implications for public health and urban resilience

**RESPONSE:**

After internal deliberations, we decided to add the following sub-headers to the DISCUSSION section:

o Changes in WASH access and behaviours during water-logging

o Socioeconomic disparities in WASH access during climate stress

o Strengths and Limitations
